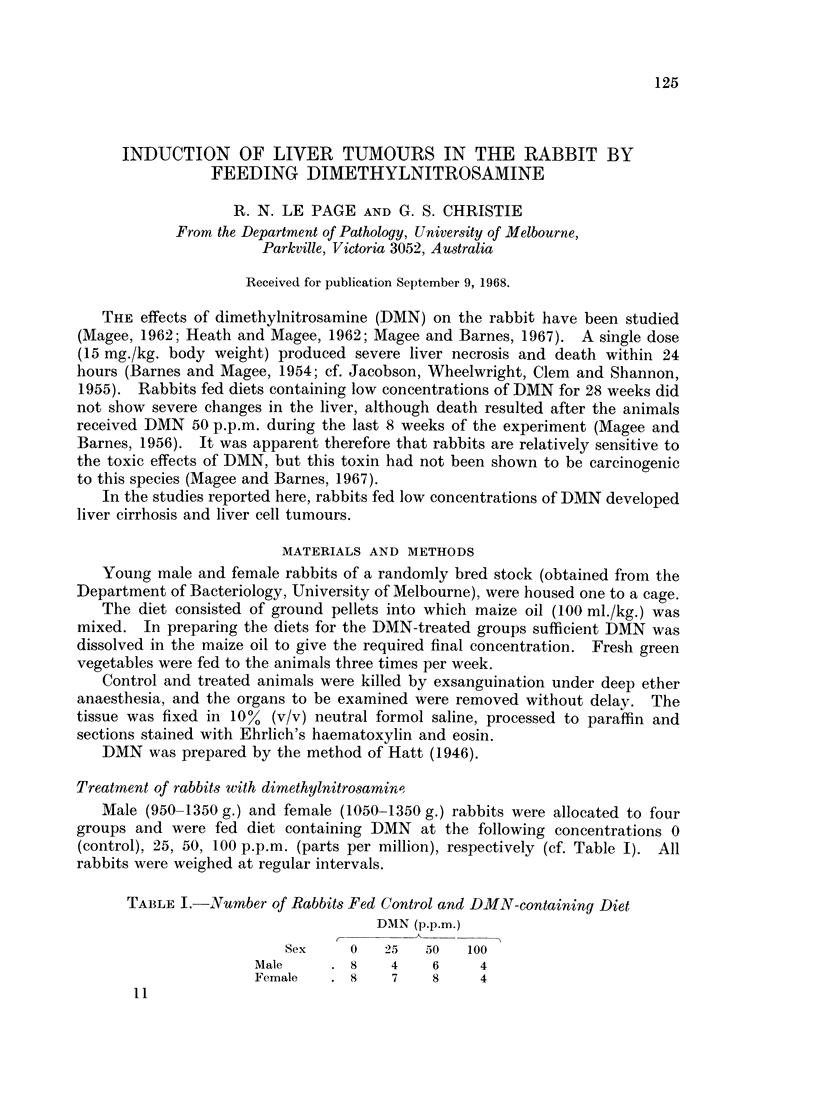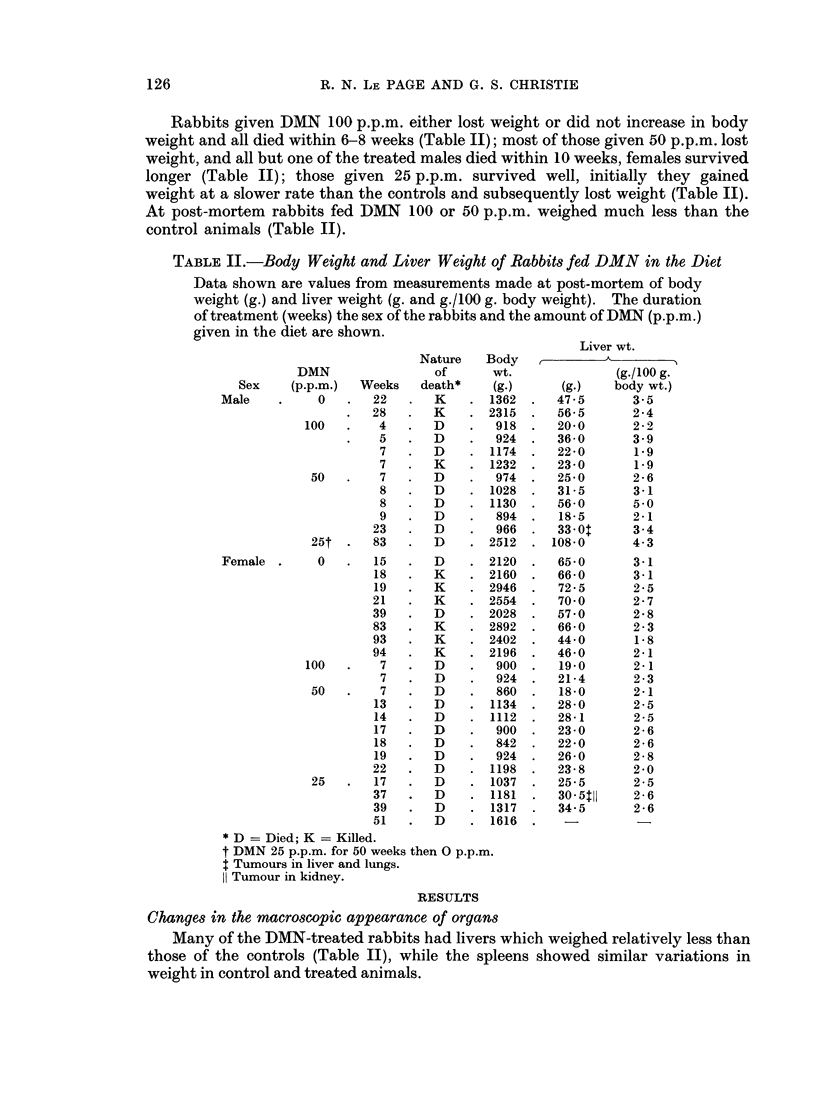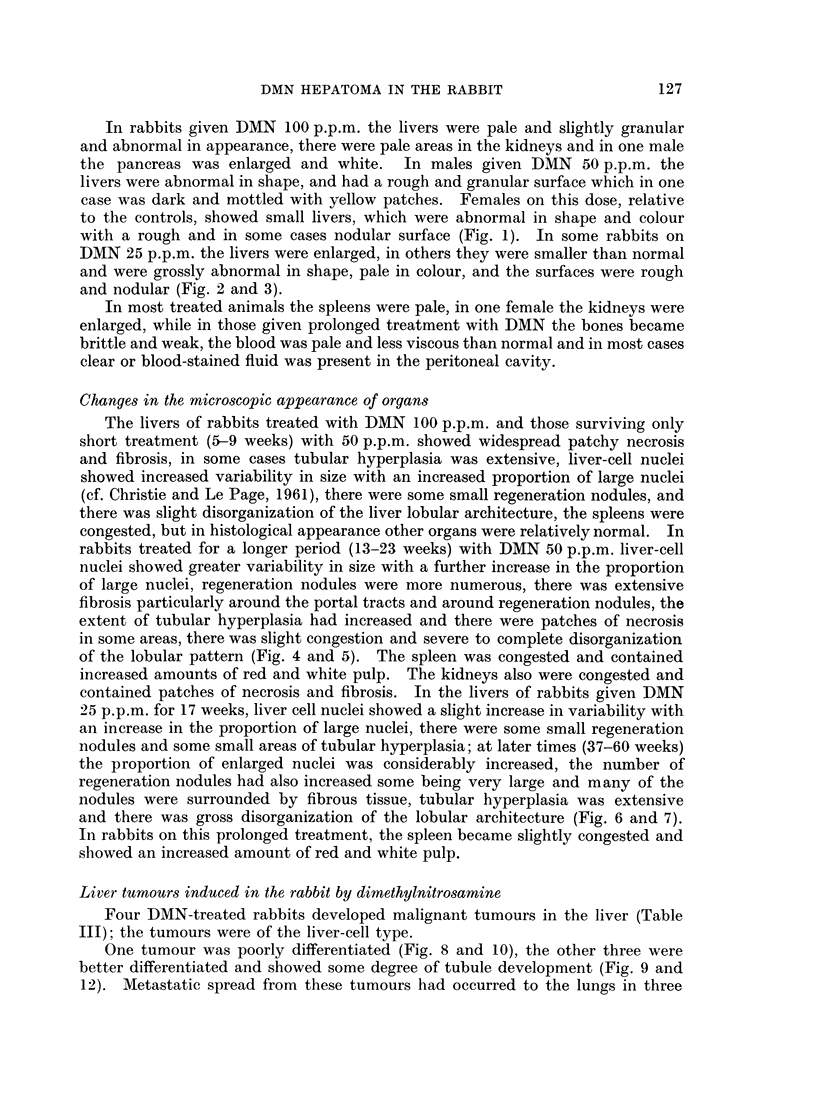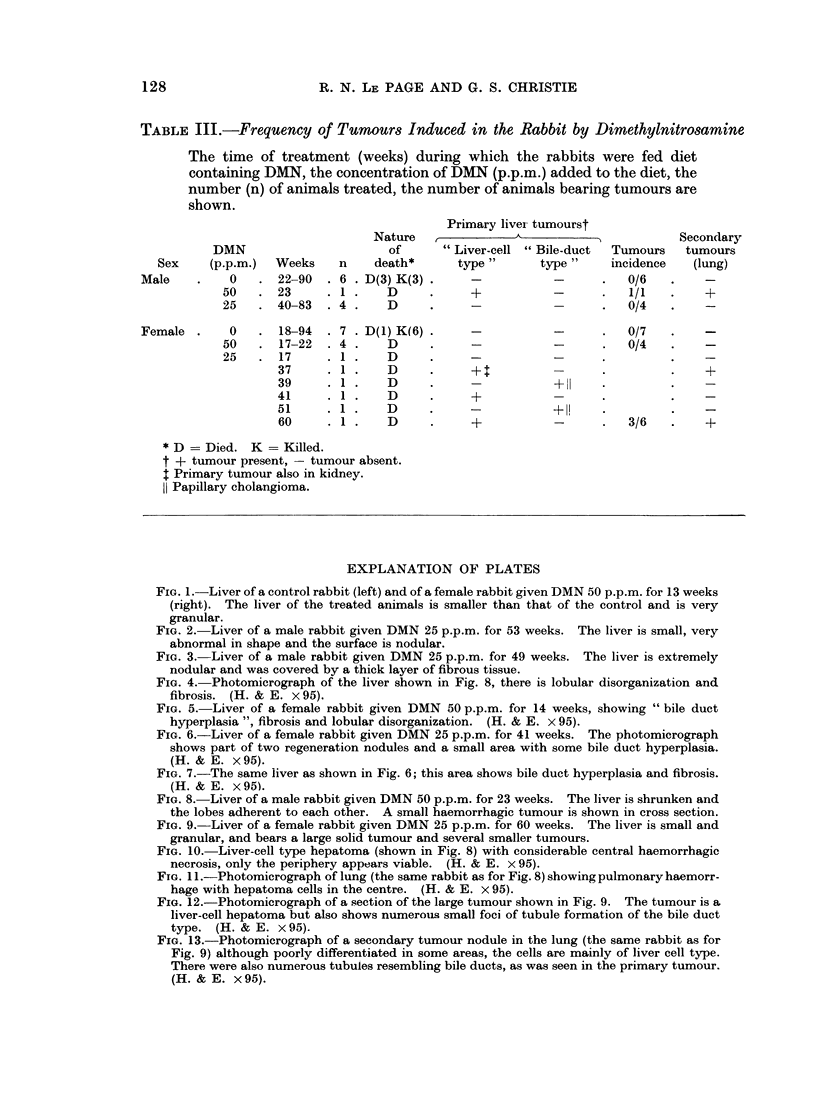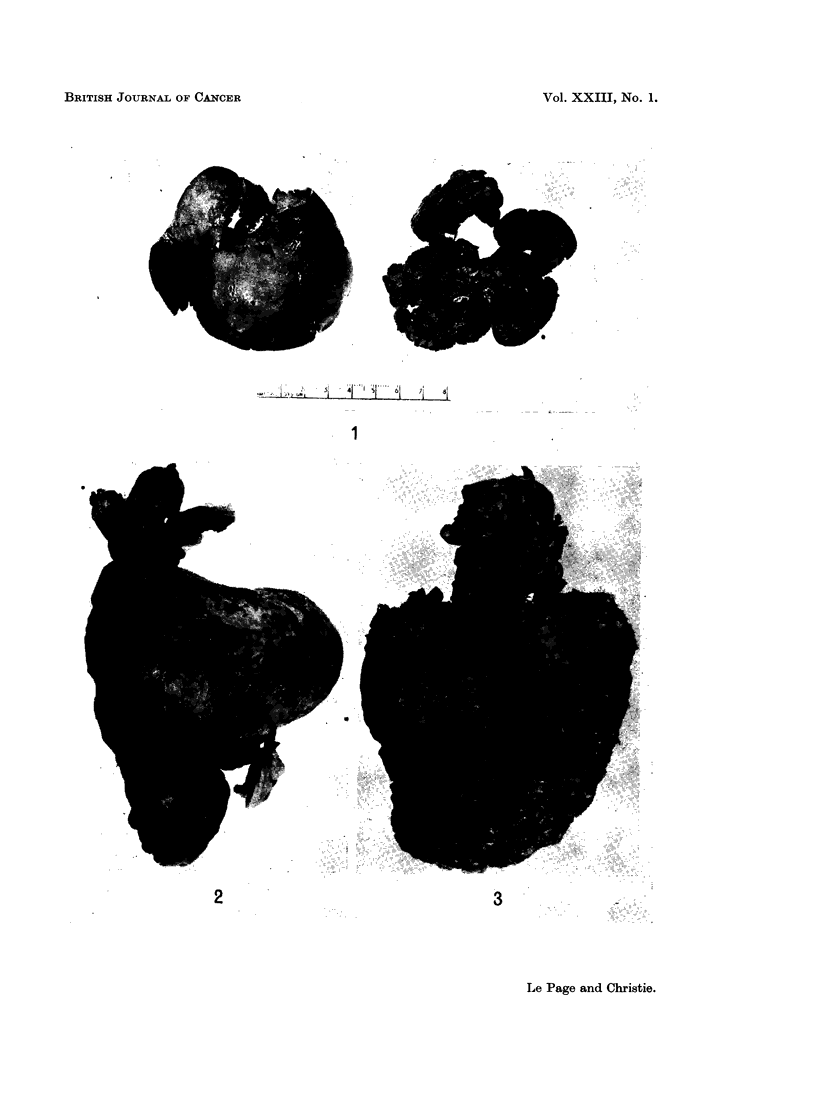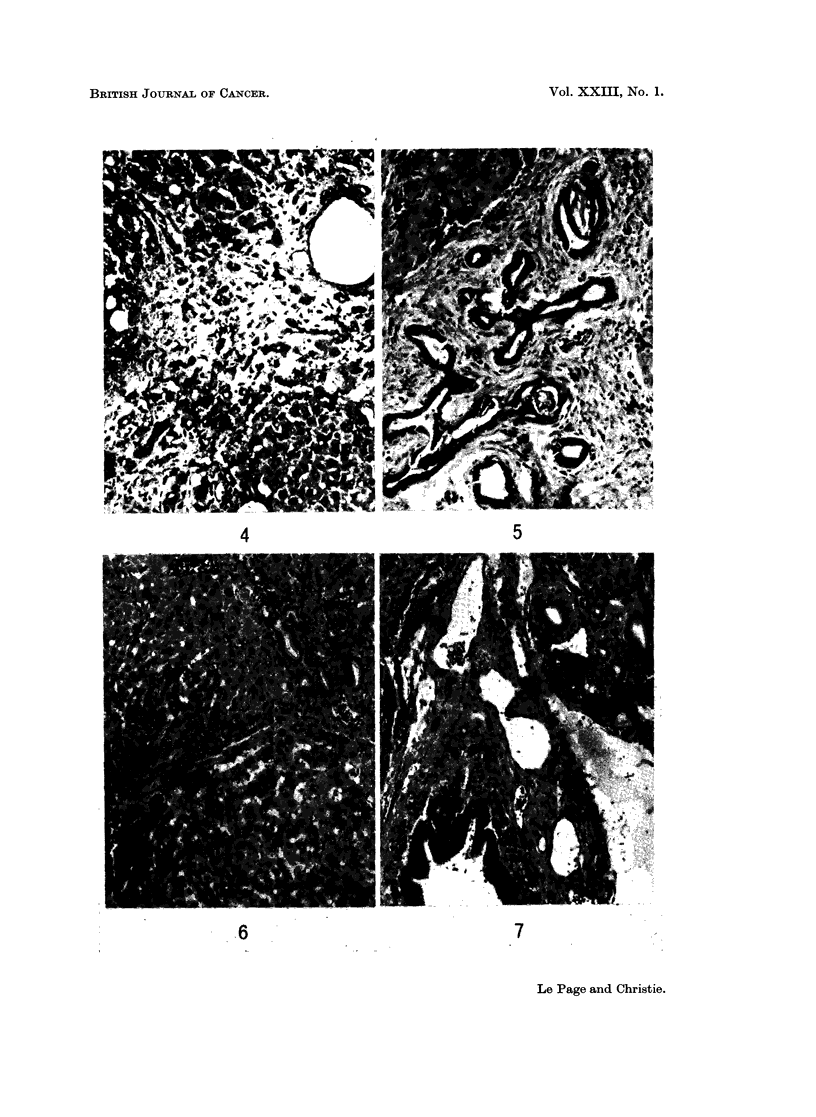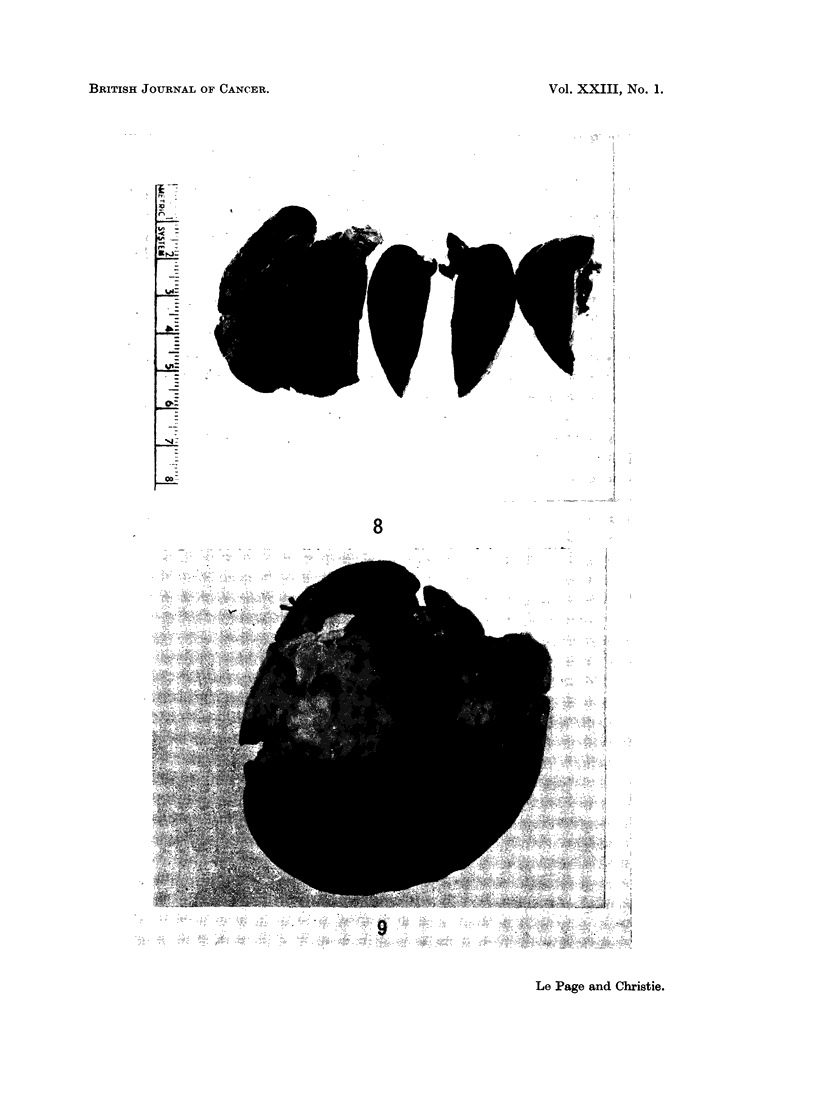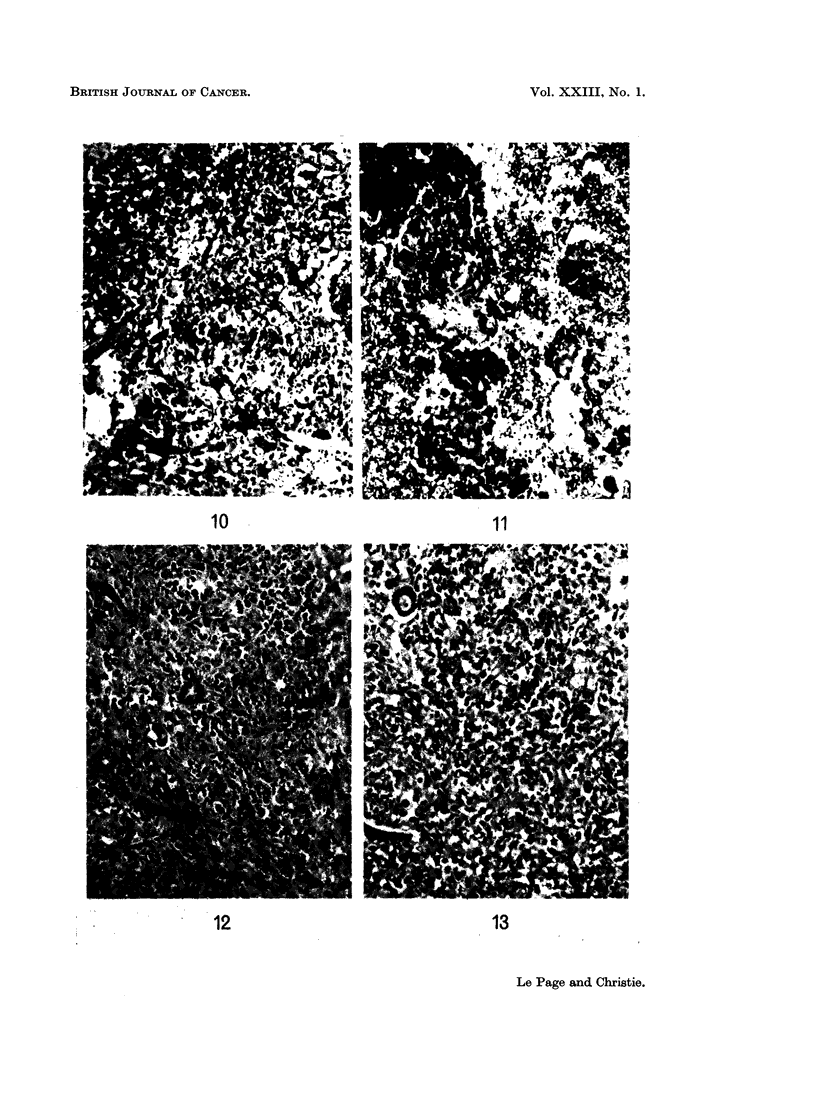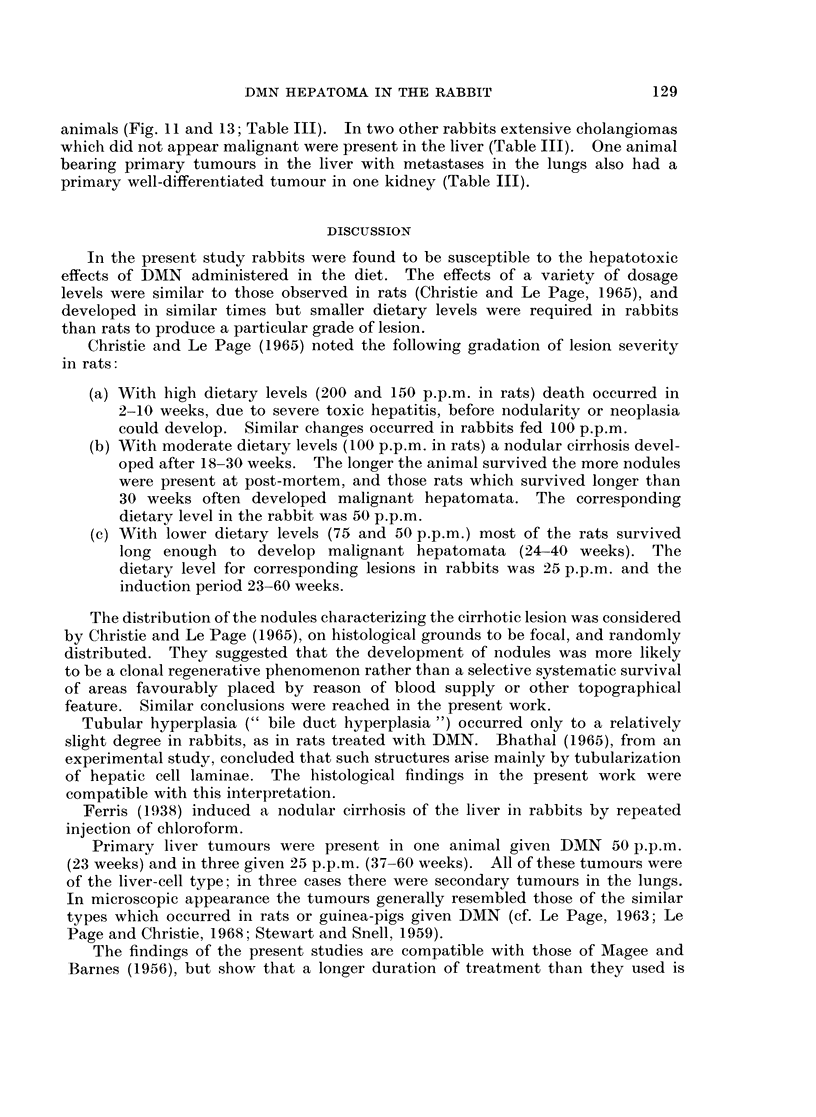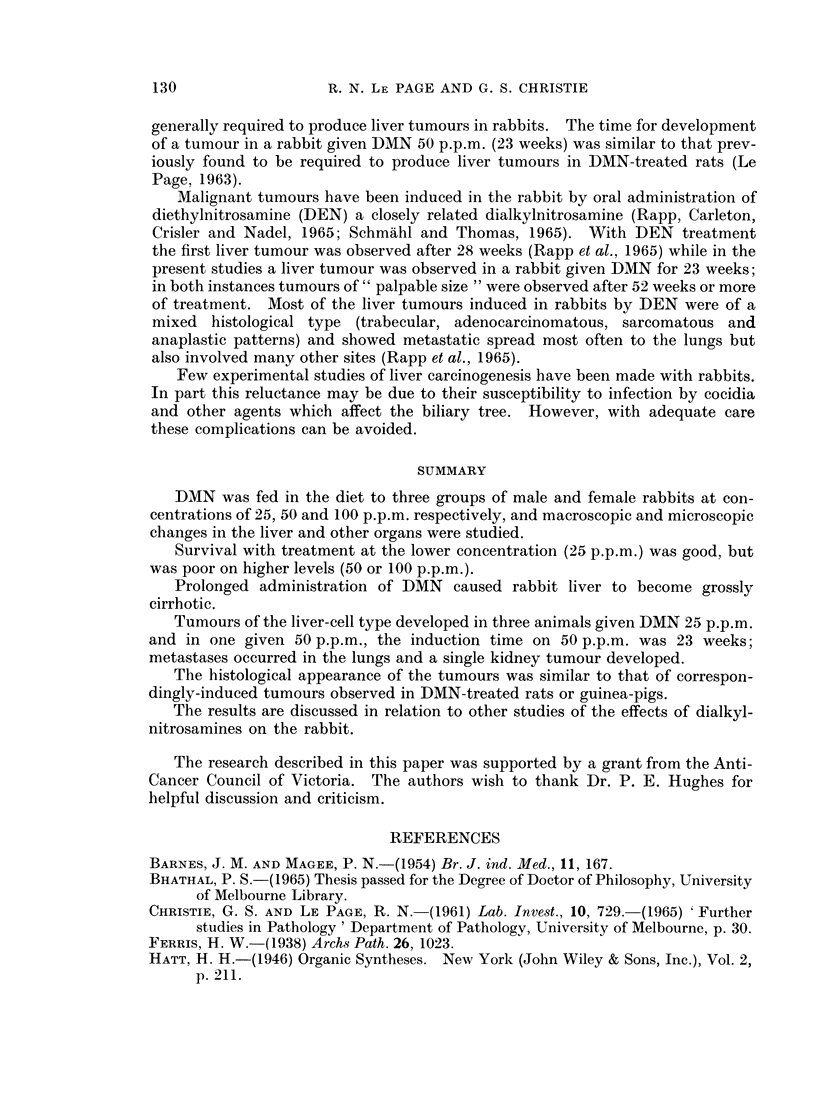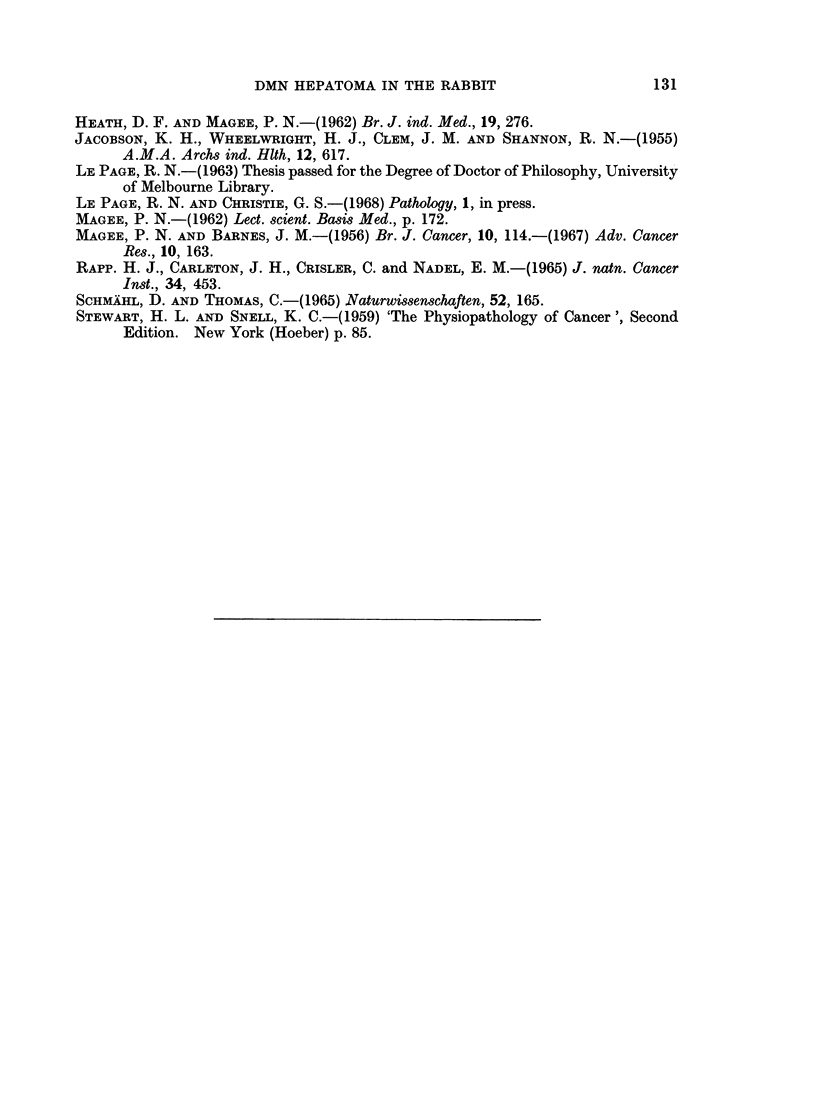# Induction of liver tumours in the rabbit by feeding dimethylnitrosamine.

**DOI:** 10.1038/bjc.1969.18

**Published:** 1969-03

**Authors:** R. N. Le Page, G. S. Christie

## Abstract

**Images:**


### INDUCTION OF LIVER TUMOURS IN THE RABBIT BY FEEDING DIMETHYLNITROSAMINE

R. N. LE PAGE AND G. S. CHRISTIE

*From the Department of Pathology, University of Melbourne, Parkville, Victoria 3052, Australia*

Received for publication September 9, 1968.

THE effects of dimethylnitrosamine (DMN) on the rabbit have been studied (Magee, 1962; Heath and Magee, 1962; Magee and Barnes, 1967). A single dose (15 mg./kg. body weight) produced severe liver necrosis and death within 24 hours (Barnes and Magee, 1954; cf. Jacobson, Wheelwright, Clem and Shannon, 1955). Rabbits fed diets containing low concentrations of DMN for 28 weeks did not show severe changes in the liver, although death resulted after the animals received DMN 50 p.p.m. during the last 8 weeks of the experiment (Magee and Barnes, 1956). It was apparent therefore that rabbits are relatively sensitive to the toxic effects of DMN, but this toxin had not been shown to be carcinogenic to this species (Magee and Barnes, 1967).

In the studies reported here, rabbits fed low concentrations of DMN developed liver cirrhosis and liver cell tumours.

### MATERIALS AND METHODS

Young male and female rabbits of a randomly bred stock (obtained from the Department of Bacteriology, University of Melbourne), were housed one to a cage.

The diet consisted of ground pellets into which maize oil (100 ml./kg.) was mixed. In preparing the diets for the DMN-treated groups sufficient DMN was dissolved in the maize oil to give the required final concentration. Fresh green vegetables were fed to the animals three times per week.

Control and treated animals were killed by exsanguination under deep ether anaesthesia, and the organs to be examined were removed without delay. The tissue was fixed in 10% (v/v) neutral formol saline, processed to paraffin and sections stained with Ehrlich's haematoxylin and eosin.

DMN was prepared by the method of Hatt (1946).

*Treatment of rabbits with dimethylnitrosamine*

Male (950–1350 g.) and female (1050–1350 g.) rabbits were allocated to four groups and were fed diet containing DMN at the following concentrations 0 (control), 25, 50, 100 p.p.m. (parts per million), respectively (cf. Table I). All rabbits were weighed at regular intervals.

TABLE I.—*Number of Rabbits Fed Control and DMN-containing Diet*

| | DMN (p.p.m.) | | | |
|---|---|---|---|---|
| Sex | 0 | 25 | 50 | 100 |
| Male | 8 | 4 | 6 | 4 |
| Female | 8 | 7 | 8 | 4 |

Rabbits given DMN 100 p.p.m. either lost weight or did not increase in body weight and all died within 6–8 weeks (Table II); most of those given 50 p.p.m. lost weight, and all but one of the treated males died within 10 weeks, females survived longer (Table II); those given 25 p.p.m. survived well, initially they gained weight at a slower rate than the controls and subsequently lost weight (Table II). At post-mortem rabbits fed DMN 100 or 50 p.p.m. weighed much less than the control animals (Table II).

TABLE II.—*Body Weight and Liver Weight of Rabbits fed DMN in the Diet*

Data shown are values from measurements made at post-mortem of body weight (g.) and liver weight (g. and g./100 g. body weight). The duration of treatment (weeks) the sex of the rabbits and the amount of DMN (p.p.m.) given in the diet are shown.

| Sex | DMN (p.p.m.) | Weeks | Nature of death* | Body wt. (g.) | Liver wt. (g.) | Liver wt. (g./100 g. body wt.) |
|---|---|---|---|---|---|---|
| Male | 0 | 22 | K | 1362 | 47·5 | 3·5 |
| | | 28 | K | 2315 | 56·5 | 2·4 |
| | 100 | 4 | D | 918 | 20·0 | 2·2 |
| | | 5 | D | 924 | 36·0 | 3·9 |
| | | 7 | D | 1174 | 22·0 | 1·9 |
| | | 7 | K | 1232 | 23·0 | 1·9 |
| | 50 | 7 | D | 974 | 25·0 | 2·6 |
| | | 8 | D | 1028 | 31·5 | 3·1 |
| | | 8 | D | 1130 | 56·0 | 5·0 |
| | | 9 | D | 894 | 18·5 | 2·1 |
| | | 23 | D | 966 | 33·0‡ | 3·4 |
| | 25† | 83 | D | 2512 | 108·0 | 4·3 |
| Female | 0 | 15 | D | 2120 | 65·0 | 3·1 |
| | | 18 | K | 2160 | 66·0 | 3·1 |
| | | 19 | K | 2946 | 72·5 | 2·5 |
| | | 21 | K | 2554 | 70·0 | 2·7 |
| | | 39 | D | 2028 | 57·0 | 2·8 |
| | | 83 | K | 2892 | 66·0 | 2·3 |
| | | 93 | K | 2402 | 44·0 | 1·8 |
| | | 94 | K | 2196 | 46·0 | 2·1 |
| | 100 | 7 | D | 900 | 19·0 | 2·1 |
| | | 7 | D | 924 | 21·4 | 2·3 |
| | 50 | 7 | D | 860 | 18·0 | 2·1 |
| | | 13 | D | 1134 | 28·0 | 2·5 |
| | | 14 | D | 1112 | 28·1 | 2·5 |
| | | 17 | D | 900 | 23·0 | 2·6 |
| | | 18 | D | 842 | 22·0 | 2·6 |
| | | 19 | D | 924 | 26·0 | 2·8 |
| | | 22 | D | 1198 | 23·8 | 2·0 |
| | 25 | 17 | D | 1037 | 25·5 | 2·5 |
| | | 37 | D | 1181 | 30·5‡‖ | 2·6 |
| | | 39 | D | 1317 | 34·5 | 2·6 |
| | | 51 | D | 1616 | — | — |

* D = Died; K = Killed.

† DMN 25 p.p.m. for 50 weeks then O p.p.m.

‡ Tumours in liver and lungs.

‖ Tumour in kidney.

### RESULTS

### *Changes in the macroscopic appearance of organs*

Many of the DMN-treated rabbits had livers which weighed relatively less than those of the controls (Table II), while the spleens showed similar variations in weight in control and treated animals.

In rabbits given DMN 100 p.p.m. the livers were pale and slightly granular and abnormal in appearance, there were pale areas in the kidneys and in one male the pancreas was enlarged and white. In males given DMN 50 p.p.m. the livers were abnormal in shape, and had a rough and granular surface which in one case was dark and mottled with yellow patches. Females on this dose, relative to the controls, showed small livers, which were abnormal in shape and colour with a rough and in some cases nodular surface (Fig. 1). In some rabbits on DMN 25 p.p.m. the livers were enlarged, in others they were smaller than normal and were grossly abnormal in shape, pale in colour, and the surfaces were rough and nodular (Fig. 2 and 3).

In most treated animals the spleens were pale, in one female the kidneys were enlarged, while in those given prolonged treatment with DMN the bones became brittle and weak, the blood was pale and less viscous than normal and in most cases clear or blood-stained fluid was present in the peritoneal cavity.

*Changes in the microscopic appearance of organs*

The livers of rabbits treated with DMN 100 p.p.m. and those surviving only short treatment (5–9 weeks) with 50 p.p.m. showed widespread patchy necrosis and fibrosis, in some cases tubular hyperplasia was extensive, liver-cell nuclei showed increased variability in size with an increased proportion of large nuclei (cf. Christie and Le Page, 1961), there were some small regeneration nodules, and there was slight disorganization of the liver lobular architecture, the spleens were congested, but in histological appearance other organs were relatively normal. In rabbits treated for a longer period (13–23 weeks) with DMN 50 p.p.m. liver-cell nuclei showed greater variability in size with a further increase in the proportion of large nuclei, regeneration nodules were more numerous, there was extensive fibrosis particularly around the portal tracts and around regeneration nodules, the extent of tubular hyperplasia had increased and there were patches of necrosis in some areas, there was slight congestion and severe to complete disorganization of the lobular pattern (Fig. 4 and 5). The spleen was congested and contained increased amounts of red and white pulp. The kidneys also were congested and contained patches of necrosis and fibrosis. In the livers of rabbits given DMN 25 p.p.m. for 17 weeks, liver cell nuclei showed a slight increase in variability with an increase in the proportion of large nuclei, there were some small regeneration nodules and some small areas of tubular hyperplasia; at later times (37–60 weeks) the proportion of enlarged nuclei was considerably increased, the number of regeneration nodules had also increased some being very large and many of the nodules were surrounded by fibrous tissue, tubular hyperplasia was extensive and there was gross disorganization of the lobular architecture (Fig. 6 and 7). In rabbits on this prolonged treatment, the spleen became slightly congested and showed an increased amount of red and white pulp.

*Liver tumours induced in the rabbit by dimethylnitrosamine*

Four DMN-treated rabbits developed malignant tumours in the liver (Table III); the tumours were of the liver-cell type.

One tumour was poorly differentiated (Fig. 8 and 10), the other three were better differentiated and showed some degree of tubule development (Fig. 9 and 12). Metastatic spread from these tumours had occurred to the lungs in three

TABLE III.—*Frequency of Tumours Induced in the Rabbit by Dimethylnitrosamine*

The time of treatment (weeks) during which the rabbits were fed diet containing DMN, the concentration of DMN (p.p.m.) added to the diet, the number (n) of animals treated, the number of animals bearing tumours are shown.

| Sex | DMN (p.p.m.) | Weeks | n | Nature of death* | Primary liver tumours† " Liver-cell type " | Primary liver tumours† " Bile-duct type " | Tumours incidence | Secondary tumours (lung) |
|---|---|---|---|---|---|---|---|---|
| Male | 0 | 22–90 | 6 | D(3) K(3) | – | – | 0/6 | – |
| | 50 | 23 | 1 | D | + | – | 1/1 | + |
| | 25 | 40–83 | 4 | D | – | – | 0/4 | – |
| Female | 0 | 18–94 | 7 | D(1) K(6) | – | – | 0/7 | – |
| | 50 | 17–22 | 4 | D | – | – | 0/4 | – |
| | 25 | 17 | 1 | D | – | – | | – |
| | | 37 | 1 | D | +‡ | – | | + |
| | | 39 | 1 | D | – | +‖ | | – |
| | | 41 | 1 | D | + | – | | – |
| | | 51 | 1 | D | – | +‖ | | – |
| | | 60 | 1 | D | + | – | 3/6 | + |

* D = Died. K = Killed.
† + tumour present, – tumour absent.
‡ Primary tumour also in kidney.
‖ Papillary cholangioma.

### EXPLANATION OF PLATES

FIG. 1.—Liver of a control rabbit (left) and of a female rabbit given DMN 50 p.p.m. for 13 weeks (right). The liver of the treated animals is smaller than that of the control and is very granular.

FIG. 2.—Liver of a male rabbit given DMN 25 p.p.m. for 53 weeks. The liver is small, very abnormal in shape and the surface is nodular.

FIG. 3.—Liver of a male rabbit given DMN 25 p.p.m. for 49 weeks. The liver is extremely nodular and was covered by a thick layer of fibrous tissue.

FIG. 4.—Photomicrograph of the liver shown in Fig. 8, there is lobular disorganization and fibrosis. (H. & E. ×95).

FIG. 5.—Liver of a female rabbit given DMN 50 p.p.m. for 14 weeks, showing " bile duct hyperplasia ", fibrosis and lobular disorganization. (H. & E. ×95).

FIG. 6.—Liver of a female rabbit given DMN 25 p.p.m. for 41 weeks. The photomicrograph shows part of two regeneration nodules and a small area with some bile duct hyperplasia. (H. & E. ×95).

FIG. 7.—The same liver as shown in Fig. 6; this area shows bile duct hyperplasia and fibrosis. (H. & E. ×95).

FIG. 8.—Liver of a male rabbit given DMN 50 p.p.m. for 23 weeks. The liver is shrunken and the lobes adherent to each other. A small haemorrhagic tumour is shown in cross section.

FIG. 9.—Liver of a female rabbit given DMN 25 p.p.m. for 60 weeks. The liver is small and granular, and bears a large solid tumour and several smaller tumours.

FIG. 10.—Liver-cell type hepatoma (shown in Fig. 8) with considerable central haemorrhagic necrosis, only the periphery appears viable. (H. & E. ×95).

FIG. 11.—Photomicrograph of lung (the same rabbit as for Fig. 8) showing pulmonary haemorrhage with hepatoma cells in the centre. (H. & E. ×95).

FIG. 12.—Photomicrograph of a section of the large tumour shown in Fig. 9. The tumour is a liver-cell hepatoma but also shows numerous small foci of tubule formation of the bile duct type. (H. & E. ×95).

FIG. 13.—Photomicrograph of a secondary tumour nodule in the lung (the same rabbit as for Fig. 9) although poorly differentiated in some areas, the cells are mainly of liver cell type. There were also numerous tubules resembling bile ducts, as was seen in the primary tumour. (H. & E. ×95).

1

2

3

Le Page and Christie.

4

5

6

7

8

9

10

11

12

13

animals (Fig. 11 and 13; Table III). In two other rabbits extensive cholangiomas which did not appear malignant were present in the liver (Table III). One animal bearing primary tumours in the liver with metastases in the lungs also had a primary well-differentiated tumour in one kidney (Table III).

### DISCUSSION

In the present study rabbits were found to be susceptible to the hepatotoxic effects of DMN administered in the diet. The effects of a variety of dosage levels were similar to those observed in rats (Christie and Le Page, 1965), and developed in similar times but smaller dietary levels were required in rabbits than rats to produce a particular grade of lesion.

Christie and Le Page (1965) noted the following gradation of lesion severity in rats:

(a) With high dietary levels (200 and 150 p.p.m. in rats) death occurred in 2–10 weeks, due to severe toxic hepatitis, before nodularity or neoplasia could develop. Similar changes occurred in rabbits fed 100 p.p.m.
(b) With moderate dietary levels (100 p.p.m. in rats) a nodular cirrhosis developed after 18–30 weeks. The longer the animal survived the more nodules were present at post-mortem, and those rats which survived longer than 30 weeks often developed malignant hepatomata. The corresponding dietary level in the rabbit was 50 p.p.m.
(c) With lower dietary levels (75 and 50 p.p.m.) most of the rats survived long enough to develop malignant hepatomata (24–40 weeks). The dietary level for corresponding lesions in rabbits was 25 p.p.m. and the induction period 23–60 weeks.

The distribution of the nodules characterizing the cirrhotic lesion was considered by Christie and Le Page (1965), on histological grounds to be focal, and randomly distributed. They suggested that the development of nodules was more likely to be a clonal regenerative phenomenon rather than a selective systematic survival of areas favourably placed by reason of blood supply or other topographical feature. Similar conclusions were reached in the present work.

Tubular hyperplasia (" bile duct hyperplasia ") occurred only to a relatively slight degree in rabbits, as in rats treated with DMN. Bhathal (1965), from an experimental study, concluded that such structures arise mainly by tubularization of hepatic cell laminae. The histological findings in the present work were compatible with this interpretation.

Ferris (1938) induced a nodular cirrhosis of the liver in rabbits by repeated injection of chloroform.

Primary liver tumours were present in one animal given DMN 50 p.p.m. (23 weeks) and in three given 25 p.p.m. (37–60 weeks). All of these tumours were of the liver-cell type; in three cases there were secondary tumours in the lungs. In microscopic appearance the tumours generally resembled those of the similar types which occurred in rats or guinea-pigs given DMN (cf. Le Page, 1963; Le Page and Christie, 1968; Stewart and Snell, 1959).

The findings of the present studies are compatible with those of Magee and Barnes (1956), but show that a longer duration of treatment than they used is generally required to produce liver tumours in rabbits. The time for development of a tumour in a rabbit given DMN 50 p.p.m. (23 weeks) was similar to that previously found to be required to produce liver tumours in DMN-treated rats (Le Page, 1963).

Malignant tumours have been induced in the rabbit by oral administration of diethylnitrosamine (DEN) a closely related dialkylnitrosamine (Rapp, Carleton, Crisler and Nadel, 1965; Schmähl and Thomas, 1965). With DEN treatment the first liver tumour was observed after 28 weeks (Rapp *et al.*, 1965) while in the present studies a liver tumour was observed in a rabbit given DMN for 23 weeks; in both instances tumours of " palpable size " were observed after 52 weeks or more of treatment. Most of the liver tumours induced in rabbits by DEN were of a mixed histological type (trabecular, adenocarcinomatous, sarcomatous and anaplastic patterns) and showed metastatic spread most often to the lungs but also involved many other sites (Rapp *et al.*, 1965).

Few experimental studies of liver carcinogenesis have been made with rabbits. In part this reluctance may be due to their susceptibility to infection by cocidia and other agents which affect the biliary tree. However, with adequate care these complications can be avoided.

### SUMMARY

DMN was fed in the diet to three groups of male and female rabbits at concentrations of 25, 50 and 100 p.p.m. respectively, and macroscopic and microscopic changes in the liver and other organs were studied.

Survival with treatment at the lower concentration (25 p.p.m.) was good, but was poor on higher levels (50 or 100 p.p.m.).

Prolonged administration of DMN caused rabbit liver to become grossly cirrhotic.

Tumours of the liver-cell type developed in three animals given DMN 25 p.p.m. and in one given 50 p.p.m., the induction time on 50 p.p.m. was 23 weeks; metastases occurred in the lungs and a single kidney tumour developed.

The histological appearance of the tumours was similar to that of correspondingly-induced tumours observed in DMN-treated rats or guinea-pigs.

The results are discussed in relation to other studies of the effects of dialkylnitrosamines on the rabbit.

The research described in this paper was supported by a grant from the Anti-Cancer Council of Victoria. The authors wish to thank Dr. P. E. Hughes for helpful discussion and criticism.

### REFERENCES

BARNES, J. M. AND MAGEE, P. N.—(1954) *Br. J. ind. Med.*, **11**, 167.

BHATHAL, P. S.—(1965) Thesis passed for the Degree of Doctor of Philosophy, University of Melbourne Library.

CHRISTIE, G. S. AND LE PAGE, R. N.—(1961) *Lab. Invest.*, **10**, 729.—(1965) ' Further studies in Pathology ' Department of Pathology, University of Melbourne, p. 30.

FERRIS, H. W.—(1938) *Archs Path.* **26**, 1023.

HATT, H. H.—(1946) Organic Syntheses. New York (John Wiley & Sons, Inc.), Vol. 2, p. 211.

HEATH, D. F. AND MAGEE, P. N.—(1962) *Br. J. ind. Med.*, **19**, 276.
JACOBSON, K. H., WHEELWRIGHT, H. J., CLEM, J. M. AND SHANNON, R. N.—(1955) *A.M.A. Archs ind. Hlth*, **12**, 617.
LE PAGE, R. N.—(1963) Thesis passed for the Degree of Doctor of Philosophy, University of Melbourne Library.
LE PAGE, R. N. AND CHRISTIE, G. S.—(1968) *Pathology*, **1**, in press.
MAGEE, P. N.—(1962) *Lect. scient. Basis Med.*, p. 172.
MAGEE, P. N. AND BARNES, J. M.—(1956) *Br. J. Cancer*, **10**, 114.—(1967) *Adv. Cancer Res.*, **10**, 163.
RAPP. H. J., CARLETON, J. H., CRISLER, C. and NADEL, E. M.—(1965) *J. natn. Cancer Inst.*, **34**, 453.
SCHMÄHL, D. AND THOMAS, C.—(1965) *Naturwissenschaften*, **52**, 165.
STEWART, H. L. AND SNELL, K. C.—(1959) 'The Physiopathology of Cancer ', Second Edition. New York (Hoeber) p. 85.